# p8/TTDA Overexpression Enhances UV-Irradiation Resistance and Suppresses TFIIH Mutations in a *Drosophila* Trichothiodystrophy Model

**DOI:** 10.1371/journal.pgen.1000253

**Published:** 2008-11-14

**Authors:** Javier Aguilar-Fuentes, Mariana Fregoso, Mariana Herrera, Enrique Reynaud, Cathy Braun, Jean Marc Egly, Mario Zurita

**Affiliations:** 1 Department of Developmental Genetics, Instituto de Biotecnología, Universidad Nacional Autónoma de México, Cuernavaca, México; 2 Institut de Génétique et de Biologie Moléculaire et Cellulaire, C.U. de Strasbourg, France; University of California San Francisco, United States of America

## Abstract

Mutations in certain subunits of the DNA repair/transcription factor complex TFIIH are linked to the human syndromes xeroderma pigmentosum (XP), Cockayne's syndrome (CS), and trichothiodystrophy (TTD). One of these subunits, p8/TTDA, interacts with p52 and XPD and is important in maintaining TFIIH stability. *Drosophila* mutants in the p52 (Dmp52) subunit exhibit phenotypic defects similar to those observed in TTD patients with defects in p8/TTDA and XPD, including reduced levels of TFIIH. Here, we demonstrate that several Dmp52 phenotypes, including lethality, developmental defects, and sterility, can be suppressed by p8/TTDA overexpression. TFIIH levels were also recovered in rescued flies. In addition, p8/TTDA overexpression suppressed a lethal allele of the *Drosophila* XPB homolog. Furthermore, transgenic flies overexpressing p8/TTDA were more resistant to UV irradiation than were wild-type flies, apparently because of enhanced efficiency of cyclobutane-pyrimidine-dimers and 6–4 pyrimidine-pyrimidone photoproducts repair. This study is the first using an intact higher-animal model to show that one subunit mutant can trans-complement another subunit in a multi-subunit complex linked to human diseases.

## Author Summary

TFIIH participates in RNA polymerase II transcription, nucleotide excision repair, and control of the cell cycle. In humans, certain mutations in the XPB and XPD subunits of TFIIH generate the syndromes trichothiodystrophy (TTD), xeroderma pigmentosum (XP), and Cockayne's syndrome (CS). In contrast, mutations in the p8/TTDA subunit have been linked only to TTD. Cells derived from TTD patients with defects in p8/TTDA have reduced levels of TFIIH. Therefore, it has been proposed that the main function of p8/TTDA is to stabilize and maintain steady-state levels of TFIIH. In *Drosophila*, mutations in *Dmp52* and *haywire* genes generate phenotypes that share similarities with those associated with mutations in their human counterparts, including reduced TFIIH levels. We report that p8/TTDA overexpression suppressed accumulated developmental defects associated with mutations in the *Dmp52* and *haywire* genes. We also provide evidence suggesting that the rescue of these defects is, in part, because of the recovery of normal TFIIH levels in mutant flies. These results indicate that overexpression of p8/TTDA trans-complemented mutations in other TFIIH subunits and suppressed defects accumulated during fly development. The overexpression of p8/TTDA in wild-type flies increased their UV irradiation resistance, apparently because of more efficient nucleotide excision repair.

## Introduction

The integrity of the DNA molecule can be disrupted by chemical and physical factors that cause diverse types of damage. The nucleotide excision repair (NER) pathway operates when DNA is damaged by the covalent addition of methyl groups, the formation of cyclobutane-pyrimidine dimers (CPDs), or the crosslinking of bases in opposite strands [1]. In eukaryotes, NER involves at least 35 proteins that participate in damaged-base recognition, oligonucleotide excision, and molecular repair. An important factor in NER is TFIIH, which also participates in basal transcription mediated by RNA polymerases I and II [2],[3]. TFIIH is a 10-protein complex composed of two subcomplexes. The subunits XPB, XPD, p62, p52, p44, p34, and p8 come together to form the core subcomplex of TFIIH, which preferentially participates in NER. The subunits cdk7, cycH, and MAT1 form the cdk-activating kinase subcomplex (CAK), which is involved in controlling the cell cycle [2]. Together, the core and CAK form the 10-protein TFIIH complex that has a fundamental role in RNA polymerase II (pol II) transcription [3]. The TFIIH complex possesses several enzymatic activities that contribute to NER, transcription, and cell cycle control: XPB and XPD, which are both ATPases and DNA helicases; cdk7, which is a kinase; and p44, which is an ubiquitin ligase [2],[4].

In humans, mutations in XPB and XPD subunits cause xeroderma pigmentosum (XP), combined Cockayne's syndrome with xeroderma pigmentosum (CS/XP), and trichothiodystrophy (TTD) [1],[5]. XP is primarily related to defects in NER, CS is associated with deficiencies in transcription-coupled repair (TCR), and TTD is linked to reduced transcription and DNA repair deficiencies [6]. XP patients have sunlight hypersensitivity, abnormal skin pigmentation, and a high predisposition for skin cancer. Individuals afflicted with CS have slow postnatal growth and exhibit defects in nervous system development. TTD patients also have nervous system defects, and have brittle hair, ichthyosis, and fragile nails [6]. A particular form of TTD, termed TTD-A, was recently linked to mutations in the p8 subunit, referred here as p8/TTDA. A characteristic of the cells derived from patients with TTD-A, and XPD-linked TTD, is a reduction in basal TFIIH levels [3]. Intriguingly, p8/TTDA seems not to be an essential gene because humans homozygous for a mutation in the start codon that may result in complete loss of the protein or a truncated peptide survive, as do yeast strains containing disruptions of the homologous gene [3],[7]. The p8/TTDA gene encodes a 72-amino acid protein that is highly conserved in all eukaryotic organisms [3],[7]. Transfection of wild-type p8/TTDA rescues TFIIH levels and the UV-sensitive phenotype in p8/TTDA and XPD-derived cultured cells, showing that p8/TTDA is essential for maintaining steady-state levels of TFIIH [8]. p8/TTDA interacts with TFIIH p52 [8],[9] and XPD subunits [8], and functions primarily in NER. XPB ATPase activity, which is required for NER, is modulated by the interaction of p8/TTDA and p52 [10]. p8/TTDA exists in two different pools, one in the cytoplasm and one in the nucleus. After DNA damage, p8/TTDA forms a more stable association with TFIIH in nuclei [11].

Recent studies have shown that the fruit fly, *Drosophila melanogaster*, is a useful model organism for the study of several human diseases. In a number of important cases, mutation or overexpression of a disease-related human gene generates an equivalent phenotype in the fly [12]. An important feature of fly models of human diseases is the ability to use such models in genetic screens to identify new mutations or modifications in gene expression that suppress defective phenotypes [13]. Interestingly, flies carrying mutations in the XPB *(haywire)* and p52 (*Dmp52*) TFIIH subunits of *Drosophila* exhibit phenotypes that are comparable to those observed in humans [14]–[17]. In addition, the neurological defects, brittle bristles phenotype, UV-irradiation hypersensitivity, and cuticle defects in flies with defects in TFIIH components appear to exhibit similarities to some symptoms of TTD individuals at the molecular level [14]–[16]. These similarities include reduced transcription of specific genes that are normally required at high levels in terminally differentiated cells [15],[16], and a reduction in TFIIH levels [16],[18]. In this work we report that overexpression of the *Drosophila* homologue of p8/TTDA (Dmp8/TTDA), suppresses lethal mutations in the *Dmp52* and *haywire* genes. Rescued flies suppress developmental defects, including brittle bristles and thin cuticle, and recover basal TFIIH levels. In addition, transgenic flies overexpressing Dmp8/TTDA are more resistant to UV irradiation than are wild-type organisms and are more efficient in the repair of cyclobutane-pyrimidine dimers (CPDs) and 6-4 pyrimidine-pyrimidone photoproducts (6-4PPs). Collectively, our results open the possibility of a therapy based on enhancement of p8/TTDA function in patients afflicted with TFIIH-related syndromes.

## Materials and Methods

### *Drosophila* Strains

The OreR *Drosophila* strain was used as a control. All *mrn and haywire* alleles used in this work have been previously characterized [15]–[17]. The parental strain for the *mrn* alleles has the *red* and *ebony* markers and was used in some UV irradiation experiments as control.

### Transgenic Flies and Rescue

The complete *Drosophila* wild-type *p8/TTDA* DNA sequence was amplified by PCR and cloned into the pCaSper*hsp83* vector and sequenced to verify its integrity. Constructs encoding six histidines at the NH-terminus (H6-*Dmp8/TTDA*) or COOH-terminus (*Dmp8/TTDA*-H6) of Dmp8/TTDA were also cloned into the pCaSperhsp83 vector. Transgenic flies were constructed using a standard microinjection protocol. The location of transgenes on different chromosomes was determined by balancer mapping. Rescue experiments were performed by crossing *pCaSper-hsp83-p8/TTDA* transgenic fly lines with *mrn* and *hay* alleles, as previously described [16]. In brief, balanced transgenic flies expressing *p8/TTDA* in the X and second chromosomes and an MKRS/TM3 third chromosome were crossed with different *mrn* and *hay* alleles balanced with TM6B. The F1 progeny were crossed to generate homozygous *mrn* or *hay* and heteroallelic *EP3605/mrn* flies containing one or two copies of the transgene in either the second or X chromosome.

### UV-Irradiation Sensitivity Assays

Third instar wild-type, rescued and transgenic larvae were irradiated at different UV-B light dosages (Joules/m^2^) using a UV Stratalinker 2400 (Stratagene). The larvae were then allowed to develop into adults and the emerged population was counted.

### Immunohistochemistry and Quantification of Fluorescence in Confocal Sections of Salivary Gland Nuclei

Third instar larvae salivary glands from rescued homozygous *mrn* mutants and heteroallelic combinations of *EP3605* and *mrn* 1, 3 and 5 alleles were dissected, immunostained and quantified as previously described [16]. Briefly, using confocal microscopy, representative images of immunostained XPD, XPB, TBP and histones in nuclear sections from wild-type and each *Dmp52* genotype were obtained. Nuclear areas (156 pixels/nucleus) were analyzed from each genotype using a photon-counting protocol. Fluorescence-intensity distribution frequencies were obtained and represented as a histogram. Relative fluorescence ratios are presented as a bar chart, which shows the average intensity of XPB/TBP, XPB/histones, XPD/TBP and XPD/histones (±standard errors) in the *y*-axis for each genotype of *Dmp52* mutants and rescued organisms (*x*-axis).

### Southwestern Dot-Blot and ELISA Assays for Measuring DNA Damage

Ten micrograms of genomic DNA isolated from third instar larvae was dotted onto a nitrocellulose membrane and probed with an anti-CPD antibody using a standard Southwestern analysis protocol (Kamiya Biomedical Company, Seattle WA). To measure 6-4PPs, we performed ELISA assays using a specific anti-6-4PP antibody following the standard protocol recommended by the supplier (Kamiya Biomedical Company).

### Western Blot Experiments

In general, total protein soluble extracts were prepared from adult flies and standardized. Then the samples were loaded in 10% SDS-PAGE gels and the proteins transferred to nitrocellulose filters. A specific anti CTD-Ser-5-P antibody was used following standard protocols.

### Purification of Recombinant TFIIH Complexes

Typically, 10^8^ cells were infected with combinations of recombinant baculoviruses expressing XPB, XPD, p62, p52 (or the various mutant versions), and p44, p34, p8, cdk7, cyclin H and MAT1 as indicated, and collected 48 h after infection. Cells were washed with phosphate-buffered saline, 30% glycerol and disrupted in 10 ml buffer A (20 mM Tris-HCl pH 7.5, 150 mM NaCl, 20% glycerol, 0.1% Nonidet P40, 5 mM b-mercaptoethanol) using a dounce homogenizer. After centrifugation at 14,000× g for 30 min at 4°C, clarified lysates were loaded onto a heparin-Ultrogel column (Sepracor) pre-equilibrated in buffer A. After extensive washing with buffer A containing 300 mM NaCl, the proteins were eluted with buffer A containing 500 mM NaCl. The eluted fractions were dialyzed for 2 h against 50 mM Tris-HCl pH 7.9, 50 mM KCl, 20% glycerol, 0.1 mM EDTA and 0.5 mM dithiothreitol, and immunopurified using the 1H5 anti-p44 antibody [19].

### Transcription and Dual-Incision NER Assays

Run-off transcription was carried out as previously described [19]. The dual-incision assay was performed according published methods [20],[21]. Briefly, repair reactions were carried out in buffer containing 45 mM HEPES pH 7.8, 70 mM KCl, 5 mM MgCl_2_, 1 mM dithiothreitol, 0.3 mM EDTA, 10% glycerol and 2 mM ATP. Each reaction contained 50 ng XPG, 20 ng XPF/ERCC1, 10 ng XPC-hHR23B, 50 ng RPA, 25 ng XPA and either 1.5 µl of HeLa TFIIH (Hep fr. IV ) or recombinant TFIIH complexes that included a wild-type or mutant p52 subunit. After pre-incubating for 10 min at 30°C, 50 ng of damaged circular template DNA containing a single 1,3-intrastrand d(GpTpG) cisplatin-DNA cross-link (Pt-GTG) was added and reactions were continued for 90 min at 30°C. The reactions were stopped by rapid freezing. After annealing with 9 ng of the complementary oligonucleotide, a mixture of [a-^32^P] dNTPs (3000 mCi/mmol) was added and residues were incorporated using Sequenase V2.1 (USB). The excised, radiolabeled fragments were separated on a 14% urea–polyacrylamide gel and visualized by autoradiography [19],[21].

## Results/Discussion

### Dmp8/TTDA Overexpression Suppresses Mutations in *Dmp52*

Our group and others have previously demonstrated that homozygous point mutations in the genes *marionette* (*mrn*), encoding the *Drosophila* homolog of p52 (*Dmp52*), and *haywire*, the *Drosophila* homolog of XPB, are lethal and share chromosomal fragility and defective developmental phenotypes [14]–[16]. In contrast, organisms with heteroallelic *Dmp52* combinations between a P-element insertion near the 5′ region of the *Dmp52* gene (*EP3605*) and *mrn* point mutations develop into adults, but are sterile, have reduced levels of TFIIH, and present with brittle bristles and cuticle deformations typical of TFIIH-deficient flies [15],[16]. As p8/TTDA overexpression rescues the UV-sensitive phenotype and TFIIH levels in XPD-deficient cultured cells [8], we tested whether overexpression of Dmp8/TTDA might rescue the larval lethality and adult phenotypic defects observed in *Dmp52* mutant flies. To answer this question, we generated transgenic flies that overexpress *Dmp8/TTDA* under the control of the *HSP83* promoter. This promoter is constitutive and drives moderate levels of transgene expression in all tissues. Transgene expression in the transgenic lines, TTDA1 and TTDA5, was verified by RT-PCR (Figure S1). Two additional transgenic lines used in this work, TTDA8 and TTDA9, express hexahistidine (H6)-tagged recombinant Dmp8/TTDA protein with the tag present at either the N-terminus (H6-Dmp8/TTDA) or C-terminus (Dmp8/TTDA-H6), respectively; the proteins can be detected by Western blotting and immunocytochemistry (Figure S1). In general, transgenic flies with only a single copy of the transgene expressed approximately 10 times more *Dmp8/TTDA* transcript than did wild-type flies. In fact, the levels of endogenous *Dmp8/TTDA* mRNA were very low and were more difficult to detect than were other TFIIH transcripts, suggesting that Dmp8/TTDA could be a limiting factor for TFIIH function (Figure S1B; unpublished observations).

**Figure 1:**
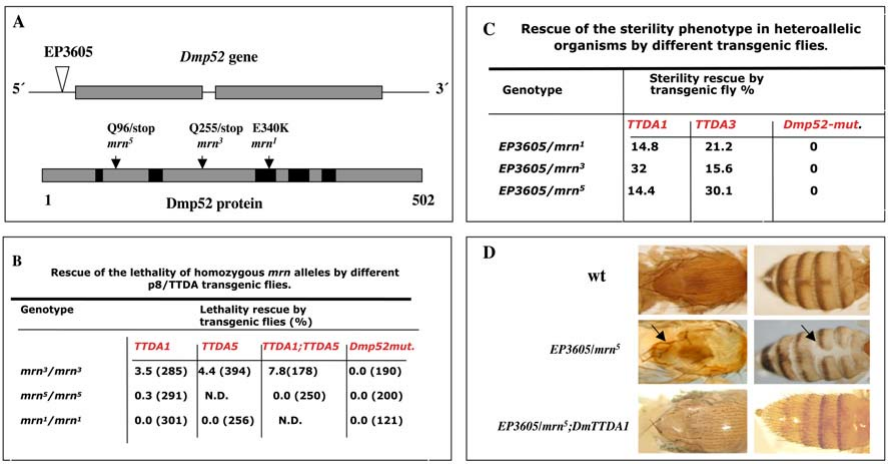
Rescue of *Dmp52 (mrn)* mutants by transgenic flies overexpressing *Dmp8/TTDA*. (A) Schematic diagrams of the *Dmp52* mutant alleles used in this work. An EP-transposable element inserted upstream of the first *Dmp52* exon is indicated as an inverted triangle. The molecular nature and positions of the previously characterized point mutations [16] are also indicated. Black boxes represent regions conserved between human and fly p52. For more details on genotypic and phenotypic characterization, see reference [16]. (B) Rescue of lethality of homozygous *mrn* alleles by *Dmp8/TTDA* transgenic fly lines. The percentage represents the number of homozygous individuals recovered relative to the number expected with full complementation (number in parenthesis). Note that two transgene copies increased the number of viable organisms. (C) Rescue of the sterility phenotype in heteroallelic organisms by different transgenic lines. The percentage represents the number of individual flies that were fertile and had progeny that developed at least to larval stage. Ten to 25 flies were tested for each condition. Homozygous *EP3605/EP3605* and heteroallelic adults (*EP3605/ mrn^1^*, *mrn^3^ and mrn^5^*) are 100% sterile. *Dmp52-mut* is a transgenic line that expresses a double point mutant (E310KR314E) [16]. (D) Rescue of brittle-bristle and cuticle-deformation phenotypes in heteroallelic *EP3605/mrn* flies. 100% of adult heteroallelic flies have cuticular and bristle defects [16]. The presence of the *Dmp8/TTDA* transgene rescues both defects in all heteroallelic adult flies examined. The genotype is indicated in each panel and data quantification is shown in Table 1.

To obtain homozygous *mrn* (*mrn/mrn*) mutants and heteroallelic *Dmp52* mutants (*EP3605/mrn)*, carrying one or two copies of the *Dmp8/TTDA* transgene, we crossed different transgenic flies with flies carrying various mutations affecting the *Dmp52* gene [ref 16; see also Figure 1A]. Figure 1B shows that a single copy of the *Dmp8/TTDA* transgene partially rescued the lethal phenotype of *mrn^3^* homozygous mutant flies. Two copies of the transgene, one on each of the X chromosome and chromosome 2 (indicated in Figure 1B as TTDA1 and TTDA5), increased the number of organisms rescued. The rescue values were low, but significant, because a transgenic line that expresses a double mutant form of Dmp52 (*Dmp52^(E340K-R344E)^*) was not able to rescue any mutant lines [ref 16; Figure 1B]. We have previously shown that this double mutant form, generated by site-directed mutagenesis, abolishes the incorporation of XPB into the 10-subunit TFIIH complex, thereby dramatically reducing transcription and NER activity [16]. In addition, a line homozygous for the *mrn^3^* allele carrying three copies of the *Dmp8/TTDA* transgene is viable and fertile. Thus, overexpression of Dmp8/TTDA was able to rescue the milder homozygous *Dmp52* lethal allele, *mrn^3^*, indicating that a 10–fold increase in the expression of Dmp8/TTDA is sufficient to achieve a partial rescue of the lethality of the *mrn^3^* allele. However, two other alleles, *mrn^1^* and *mrn^5^*, which are more deleterious than *mrn^3^* [16], were not rescued (Figure 1B).

**Figure 2:**
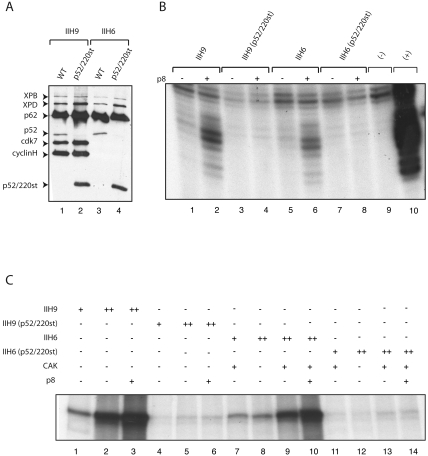
A truncated human p52 peptide (Q202/stop) equivalent to the fly *mrn^3^* allele can assemble with other TFIIH subunits. The human equivalent of the *mrn^3^* allele was co-expressed with the remaining TFIIH subunits in insect cells using the baculovirus system and affinity purified using an anti-p44 subunit antibody [19]. (A) Immunopurified recombinant TFIIH complexes (immunoprecipitated by a p44 antibody) containing either wild-type p52 or mutant p52 analyzed by Western blotting using antibodies against XPB, p62, p52, cyclin H and cdk7. The p52[Q202/stop] truncated polypeptide is indicated in the figure. IIH9 denotes a complex containing all TFIIH components except p8; IIH6 denotes the TFIIH core complex minus p8. Note that the p52/220-stop truncated polypeptide is incorporated into the TFIIH complex and allows incorporation of the XPB subunit. (B) Analysis of NER using dual-incision assays. Reaction mixtures contained recombinant XPC-HR23b, XPA, RPA, XPG and ERCC1-XPF factors with IIH9 and IIH6 complexes in the presence or absence of p8 (+ or −); closed circular plasmid DNA containing a single Pt-GTG-DNA cross-link was as a template. The lane denoted by “−” is the DNA template without TFIIH and the lane denoted by “+” contains purified human TFIIH. (C) Reconstituted transcription assay reaction mixtures contained recombinant TFIIA, TFIIB, TFIIF, TBP, TFIIE factors and purified RNA pol II with IIH9 and IIH6 complexes in the absence or in presence of p8; the adenovirus major late promoter was used as a template. Interestingly, the presence of p8 enhanced transcription by the wild-type TFIIH complex.

The *mrn^3^* allele generates a truncated peptide of 255 amino acids that contains the NH_2_-terminal portion of the protein. Interestingly, a human version of the *mrn^3^* allele co-expressed with the other recombinant human TFIIH subunits in insect cells is assembled into 6- or 9-subunit complexes (Figure 2A, indicateted as p52/220st) and allowed the incorporation of the XPB subunit into TFIIH, one of the functions of p52 (Figure 2A, lanes 2 and 4). However, these complexes lacked DNA repair and transcription activity in *in vitro* biochemical assays, both in the presence and absence of Dmp8/TTDA (Figure 2B, lanes 4 and 8; Figure 2C, lanes 6 and 13), suggesting that suppression of the *mrn^3^* allele in the fly by overexpressed Dmp8/TTDA requires a specific *in vivo* context that is not readily reconstituted *in vitro* (see below). Because p8/TTDA also interacts with the XPD component of TFIIH [8], it is possible that Dmp8/TTDA overexpression *in vivo* may stabilize the partially functional TFIIH complexes containing the Dmp52 truncated peptide through its interaction with XPD. It is also possible that in the *in vivo* context, a region of the complex present in the truncated Dmp52 protein may still interact with Dmp8/TTDA.

An initial *in vitro* analysis of the contribution of p8/TTDA to TFIIH activity showed that p8/TTDA participates in NER, but not in transcription [8]. However, it is worth noting that in *in vitro* transcription assays, Dmp8/TTDA stimulated RNA synthesis of wild-type IIH9 or in the presence of II6+CAK ∼2-fold (Figure 2C, lanes 3 and 10). The difference between the results presented here and previous reports identifying p8/TTDA as a repair-specific TFIIH subunit [8] may be attributable to differences in the TFIIH preparations [8]. Indeed, this result is in agreement with the discovery of TFB5 (the p8 yeast homologue) as component of the transcription Pre-Initiation-Complex [7]. In the yeast system, it has also been demonstrated that nuclear extracts made from yeast carrying a deletion of the TFB5 gene were deficient in transcription in vitro, and this mutant is also unable to activate transcription of inducible genes in vivo [7]. Our results suggest that the human p8/TTDA also increases transcription *in vitro* through an interaction with TFIIH, possibly by stabilizing TFIIH, however, the mechanism by which p8/TTDA enhances transcription *in vitro* still requires further study.

Heterozygous *mrn^3^/+* flies are viable, but are more sensitive to UV irradiation than are wild-type organisms (16; Figure S2). Intriguingly, the UV-irradiation sensitivity of rescued homozygous flies (TTDA/TTDA; *mrn^3^/mrn^3^*) was similar to that of heterozygous *mrn^3^/+* organisms, compared to the parental strain with the same genetic markers (Figure S2), indicating that although lethality was suppressed by Dmp8/TTDA overexpression, the NER defects of these flies were not completely restored to homozygous wild-type levels. Therefore, *mrn^3^/mrn^3^* homozygous mutant flies rescued by Dmp8/TTDA overexpression behaved like organisms that contain a single wild-type copy of *Dmp52*, and thus NER is only partially recovered.

We also found that the *Dmp8/TTDA* transgenes were able to rescue the sterility phenotype of heteroallelic *Dmp52* flies, but the mutant *Dmp52* transgene was not (Figure 1C). In all of the heteroallelic combinations tested (*EP3605/mrn^1^*, *mrn^3^*, *or mrn^5^*), sterility was suppressed to different degrees by the *Dmp8/TTDA* transgene (Figure 1C). In addition, the brittle bristle and cuticle deformation phenotypes commonly observed in the mutated heteroallelic *EP3605/mrn* flies were also suppressed. Figure 1D shows that *EP3605/mrn^5^* heteroallelic flies have defective bristles and a thin thorax and abdomen. These phenotypes were expressed in nearly 100% of these heteroallelic (*EP3605/mrn^1^*, *mrn^3^*, or *mrn^5^*) flies (Figure 1D; Table 1) [16]. However, the presence of a single extra copy of the Dmp8/TTDA transgene (denoted as *EP3605/mrn5*; DmTTDA in Figure 1D) completely suppressed these phenotypes in all flies analyzed (Figure 1D; Table 1). We have previously demonstrated that these two phenotypes are caused by deficient transcription during fly development and appear to be counterparts of the brittle hair and ichthyosis defects observed in TTD patients, which are also caused by transcriptional deficiencies in TFIIH [15]. Collectively, these results demonstrate that overexpression of Dmp8/TTDA rescues these developmental defects in a complex organism.

**Table 1:** Suppression of brittle bristle and cuticle defects by overexpression of Dmp8/TTDA in heteroallelic *Dmp52* mutant flies.

Genotype	Survival rate^a^	Brittle bristles^b^	Cuticle deformations^b^
*+/+; mrn^5^/EP3605 and +/y; mrn^5^/EP3605*	*4.9% (4/81)*	*100% (4/4)*	*100% (4/4)*
*TTDA1/TTDA1; mrn^5^/EP3605 and TTDA1/+; mrn5/EP3605*	*15.9% (21/132)*	*19% (4/21)*	*14.2% (3/21)*
*TTDA1/y; mrn^5^/EP3605*	*15.3% (15/98)*	*15.3% (15/98)*	*20% (3/15)*

a The percentages represent the heteroallelic flies without the balancers for the 3^rd^ chromosome. *+/+*; *mrn5/EP3605* flies are semi-lethal and the presence of the transgene increases the their survival rate and rescues their bristles and cuticle defects. A partial rescue of fertility is also observed (Figure 1C). The number in parenthesis is the number of heteroallelic individuals without balancers divided by the total number of flies expected for full complementation. The crosses to obtain heteroallelic +/+; *mrn^5^/EP3605* flies were independent of transgenic fly crosses.

b The percentage represents the number of adult flies with brittle bristles and cuticle defects observed in heteroallelic flies with and without the transgene. The number in parenthesis is the number of heteroallelic individuals with the mutant phenotype divided by the number of total heteroallelic flies that survive without the balancers. Brittle bristles and cuticle deformation phenotypes are shown in Figure 1D.

### Dmp8/TTDA Overexpression Suppresses *haywire* Mutants

**Figure 3:**
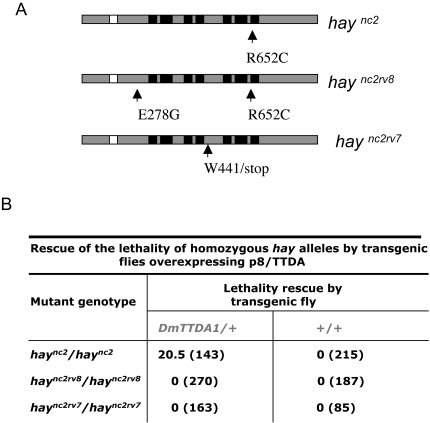
Rescue of *haywire* mutants by transgenic flies overexpressing Dmp8/TTDA. (A) Schematic diagrams of the different *hay* (*DmXPB*) mutant alleles used in this work. The molecular characterization of the mutants has been previously described [17]. *hay^nc2^* and *hay^nc2rv8^* alleles are conditional mutations that are homozygous lethal at the non-permissive temperature (25°C). *hay^nc2rv7^* generates a truncated Hay protein. The black boxes represent the helicase motifs and the white box the ATPase domain. (B) The rescue of homozygous *hay* alleles lethality by a *Dmp8/TTDA* transgene is show in table format. The percentage represents the number of homozygous individuals recovered relative to that expected with full complementation (number in parenthesis).

In addition to testing the ability of Dmp8/TTDA to suppress *Dmp52* mutants, we also determined whether overexpression of Dmp8/TTDA might be able to suppress the homozygous lethal phenotype associated with alleles of *haywire* (*hay*), which encodes for the XPB-homologous gene. To address this, we used the conditional *hay^nc2(R652C^* ^)^ and *hay^nc2rv8(R652C/E278G)^* alleles, which are lethal at 25°C, and the *hay^nc2rv7(W441stop)^* lethal allele [14],[15],[17] (Figure 3A). Genetic crosses were performed to obtain flies homozygous for the *hay^nc2^*, *hay^nc2rv8^*, and *hay^nc2rv7^*alleles [14],[15],[17], and which contained one extra copy of Dmp8/TTDA and were capable of growth at 25°C. We found that, in this genotypic context, Dmp8/TTDA overexpression rescued viability in the *hay^nc2^* flies, but not in *hay^nc2rv8^* or *hay^nc2rv7^* flies (Figure 3B). The *hay^nc2rv8^* allele encodes a Hay protein containing two point mutations, and the *hay^nc2rv7^* mutant generates a truncated Hay protein (Figure 3A); both alleles are more deleterious than is the *hay^nc2^* allele [14],[15],[17]. Interestingly, an equivalent *hay^nc2^* mutation introduced into the human XPB, has reduced transcription and repair activities and its interaction with p52 is weakened (our own unpublished results). These results indicate that even though p8/TTDA does not interact directly with XPB, p8/TTDA overexpression can suppress milder mutations in the DmXPB homologue in *Drosophila*, probably by stabilizing the interaction between XPB and p52. All thogheter these results are in agreement with a previous report that p8/TTDA overexpression can restore TFIIH levels in TTD-XPD human cells cultured *in vitro* [8], and suggest that p8/TTDA overexpression can suppress mutations in different TFIIH subunits.

### Dmp8/TTDA Overexpression Recovers Basal Levels of TFIIH in *Dmp52* Mutant Flies

**Figure 4:**
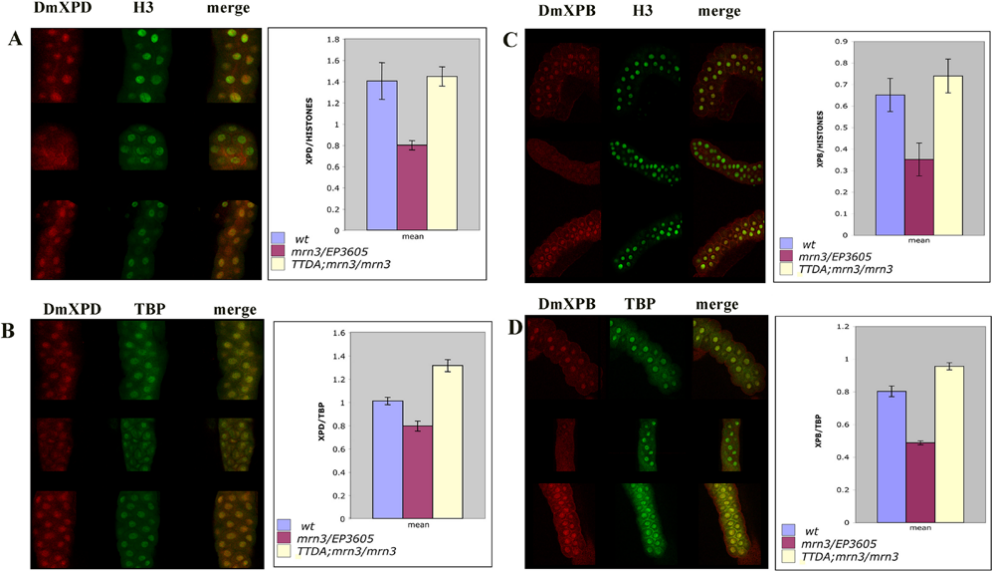
Dmp8/TTDA overexpression recovers XPD and XPB levels in *Dmp52* mutant flies. Salivary glands from third instar larvae of wild-type, heteroallelic *EP3605/mrn^3^* and homozygous *mrn^3^* flies with two copies of the *Dmp8/TTDA* transgene in chromosome 2 (TTDA/+; *mrn^3^/mrn^3^*) were dissected and co-immunostained with H3 and XPD anti-antibodies (panel A), TBP and XPD antibodies (panel B), H3 and XPB antibodies (panel C) or XPB and TBP antibodies (panel D). XPD/H3, XPD/TBP, XPB/H3 and XPB/TBP ratios were calculated using nuclei from at least ten salivary gland cells in each condition. The error bars indicate standard errors of the means.

The *EP3605* allele was generated by a transposable EP insertion in the non-coding 5′ region of the *Dmp52 gene* [16]. *EP3605* is a hypomorphic allele that may generate low levels of functional Dmp52 [16]. Accordingly, the ability of Dmp8/TTDA overexpression to rescue lethality, fertility defects, brittle bristles, and cuticle deformations in heteroallelic combinations and homozygous *mrn^3^* flies might be explained in terms of an increase in TFIIH basal levels, which are normally low in these mutants. To test this hypothesis, we performed immunofluorescence experiments using antibodies against the XPD and XPB TFIIH subunits in salivary gland nuclei of wild-type *Dmp52* heteroallelic mutants and *Dmp52* homozygous mutants carrying the *Dmp8/TTDA* transgenes. TBP and histone H3 antibodies served as internal controls. As predicted, basal XPB and XPD levels were reduced in the *Dmp52* mutant cells (Figure 4, denoted as mrn3/EP3605 in all panels); however, when Dmp8/TTDA was overexpressed in the rescued *mrn^3^/mrn^3^* homozygous line, XPD and XPB levels were restored (Figure 4, denoted as TTDA/TTDA; mrn3/mrn3). The recovery of basal XPB levels in the rescued flies is of particular importance because p52 is required for the correct assembly of XPB into TFIIH [10],[16],[19],[22], indicating the presence of more stable TFIIH complexes. These results suggest that Dmp8/TTDA overexpression increases the stability of TFIIH in *Dmp52* mutants sufficiently to allow adequate TFIIH function throughout fly development. This is of particular interest as cells derived from patients with defects in p8/TTDA and some with XPD mutations also have low basal TFIIH levels. In addition, it has been reported that the main molecular function of p8/TTDA is to control the steady state levels of TFIIH [8],[11]. Our data support this hypothesis and suggest that this is the main mechanism that allows the rescue of mutations in other TFIIH subunits by the overexpression of p8/TTDA.

### Dmp8/TTDA Overexpression Enhances UV-Irradiation Resistance in *Drosophila*

**Figure 5:**
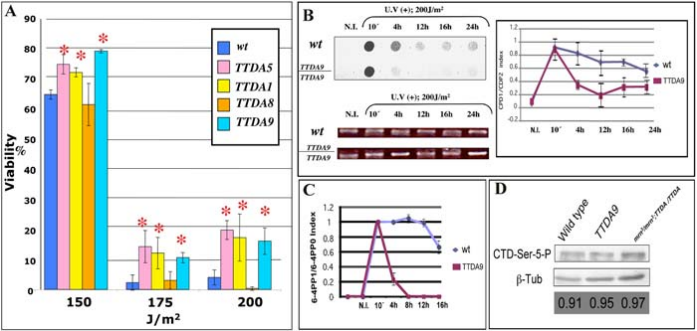
Viability, CPD and 6,4-PP repair rates, and CTD phosphorylation analysis in *Dmp8/TTDA* transgenic and wild-type lines after exposure to different doses of UV-B irradiation. (A) Third instar larvae were irradiated and then allowed to develop to adults. Survival rate is indicated for each strain. The graph represents the results of at least three independent dose-response experiments for each genotype. The statistical analysis by ANOVA indicates a P value<0.001 for the more UV-resistant transgenic lines (TTDA5, TTDA1 and TTDA9) compared to the wild-type strain. The asterisks indicate significant differences compared to the wild type. Transgenic lines overexpressing Dmp8/TTDA are denoted as TTDA1, 5, 8 and 9. (B) Representative Southwestern dot-blot analysis using an anti-CPD antibody against genomic DNA extracted at different times from wild-type and *Dmp8/TTDA* transgenic third instar larvae after UV-B irradiation at 200 J/m^2^. N.I. denotes non-irradiated DNA. The quantification of three independent Southwestern experiments is represented as a plot in which the average signal value for each sample at different times is shown relative to the signal value obtained 10 min after UV irradiation (normalized to one). The amount of DNA loaded on each dot blot was visualized in ethidium bromide stained agarose gels. Different times are indicated. (C) Quantification of 6-4PPs by ELISA assays using an anti-6-4PP–specific antibody. Three independent DNA samples derived from wild-type and transgenic third instar larvae irradiated at 200 J/m^2^ were analyzed in a typical ELISA assay. The average for each sample value is shown as the signal at different times relative to the value of the signal obtained 10 min after UV irradiation (normalized to one). (D) CTD-Ser-5 phosphorylation levels in different *Drosophila* lines. Total protein extracts from wild type, transgenic and rescued flies were analyzed in western blot experiments using an antibody that recognize the phosphorylated Serine 5 at the CTD domain of the RNA polymerase II large subunit (indicated in the figure as CTD-Ser-5-P). As internal loading control an anti-β-tubulin antibody was used. The phenotypes are indicated in the figure panel and the ratio between the CPD-Ser-5-P and β-tubulin signals are indicated at the bottom.

Enhanced stability of the TFIIH complex may also lead to an increase in TFIIH DNA repair activities. To test this hypothesis, we exposed wild-type and *Dmp8/TTDA*-overexpressing transgenic fly lines to different doses of UV radiation. We used three transgenic lines that have a single extra copy of *Dmp8/TTDA* (TTDA1, TTDA8, and TTDA9), and one line that contains three copies (TTDA5-3). As shown in Figure 5A, two lines with one copy of *Dmp8/TTDA* (lines TTDA1and TTDA9) and the line containing three copies were significantly more resistant to UV irradiation than were wild-type flies or the TTDA8 line. Although lethality was high in both transgenic and wild-type flies, survival was 3- to 4–fold higher in transgenic flies; differences were even more dramatic at higher doses (175 and 200 J/m^2^) (Figure 5A). These responses were very reproducible and a statistical analysis showed that the differences between wild-type and transgenic flies were significant (see legend to Figure 5A). Interestingly, UV resistance dropped from approximately 65% survival following irradiation at 150 J/m^2^ to 5% at 175 UV J/m2 (Figure 5A and Figure S2). This phenomenon was reproducible in different *Drosophila* strains [16; this work]. It is possible that the DNA damage produced at 175 J/m^2^ is beyond a critical repair-capacity threshold limit, thus activating check-point systems that prevent the organism (in this case third instar larvae) from continuing the developmental process.

The increase in UV-irradiation resistance suggests that overexpression of the TFIIH subunit, Dmp8/TTDA, increases NER efficiency *in vivo*. To confirm this, we analyzed the rate at which CPDs and 6-4PPs were repaired in wild-type flies and in transgenic lines that overexpressed Dmp8/TTDA. Third instar larvae were irradiated at 200 J/m^2^ and, at different times, CPDs and 6-4PPs in total purified DNA were quantified by Southwestern dot-blot analysis (CPDs) and ELISA assays (6-4PP) using antibodies specific to CPDs and 6-4PPs. After UV irradiation for 10 min, the levels of CPDs were similar in the wild-type and transgenic flies (representative dot blot and summary data obtained from quantification of three independent assays are shown in Fiure 5B). However, 4 h after irradiation, the amount of CPDs was significant lower in flies that overexpressed Dmp8/TTDA compared to wild-type flies, a difference that was maintained over time (Figure 5B).

We also observed that 6-4PPs were removed more rapidly in the transgenic flies (Figure 5C). In this case, we used ELISA assays instead of Southwestern blots because the results were more reproducible. In Figure 5C, we show an average obtained from three independent measures using the same DNA used for CPDs analysis. A dramatic difference in 6-4PP removal between the transgenic and wild-type flies is evident (Figure 5C). Thus, *Dmp8/TTDA* transgenic flies removed CPDs and 6-4PPs faster than did wild-type flies, and this increased repair rate correlated with increased resistance to UV irradiation.

It is worth noting that the rate of CPD removal in transgenic flies measured here is faster than that seen in some reports using mammalian cells [23],[24],[25]. However, another study using our technique in mammalian cells demonstrated a significant removal of CPDs 3 h after UV irradiation of wild-type cells [26], a time course that is similar to that reported here. It is also important to take into account the fact that most mammalian cell studies have employed cultured fibroblasts to measure CPD removal. To the best of our knowledge, ours is the first study to apply this technique to third instar *Drosophila* larvae, which are different from *in vitro*-cultured cells in many respects, one of which is the presence of a thick cuticle that protects the organism and necessitates the use of higher UV doses to produce damage. Another important point to consider is that at the moment of irradiation, these larvae are not only growing but are also preparing to undergo metamorphosis; it possible that the rate of DNA repair could be different during other developmental stages.

Intriguingly, the removal of 6-4PPs was very slow in wild-type organisms, only after 16 hrs after irradiation removal was ovserved, however it was very fast in flies overexpressing Dmp8/TTDA. This result is different from a previous report on the rate of 6-4PP repair in cultured *Drosophila* cells [27]. However, the methods used to measure 6-4PPs were different; in our case, we used the entire organism instead of *in vitro*-cultured cells and found a similar rate of removal in three independent DNA preparations.

The role of TFIIH in NER is to open the double-stranded DNA at the site of damage, a function that depends on the 5′-3′ helicase and ATPase activities of the XPD and XPB subunits, respectively [10]. p52 is required to incorporate XPB into the core of TFIIH and, together with p8/TTDA, may positively regulate XPB-ATPase activity [10]. TFIIH is also important in the recruitment and stabilization of several components of the NER machinery at the DNA-damaged site [22]. In addition, a stable TFIIH complex is an important prerequisite for interaction with specific factors in NER and transcription [28],[29]. It is possible that p8/TTDA overexpression may help to stabilize TFIIH, thereby enhancing some of TFIIH NER functions. In agreement with this hypothesis, an increase in p8/TTDA levels has been shown to enhance TFIIH repair activity *in vitro* [8].

Considering the *in vitro* transcription experiments presented in Figure 2 in light of the fact that overexpression of Dmp8/TTDA enhanced NER in *Drosophila*, we investigated whether overexpression of Dmp8/TTDA affected transcription in the transgenic flies. To address this question, we measured the levels of Ser-5 phosphorylation in the large subunit of the RNA pol II C-terminal domain (CTD). As has been previously established, the phosphorylation of Ser-5 in the RNA pol II large subunit CTD is a direct measure of the transcriptional activity of TFIIH [30]. To measure Ser-5 phosphorylation levels, we used an antibody that specifically recognizes this modification and protein extracts from wild-type, *Dmp8/TTDA*-transgenic flies, and rescued flies. Interestingly, CTD Ser-5 phosphorylation levels in the rescued flies are at similar levels as in wild type organisms, when the ratio between the β-tubulin levels and CTD-Ser-5 phosphorylated is compared in each phenotype (bottom of Figure 5D). However, there was no significant increase in the phosphorylation of the CTD in transgenic flies with a wild-type background (Figure 5D). These results suggest that Dmp8/TTDA is not a limiting factor in transcription.

### Concluding Remarks

There have been reports that a mutant in one subunit of a multifunctional complex can be trans-complemented by overexpression of another subunit, but only in cultured cells or in unicellular organisms [8],[31],[32]. In this work, we show that overexpression of Dmp8/TTDA can suppress a mutation in other TFIIH subunits and enhance UV-irradiation resistance in a living multicellular organism. Notably, developmental defects that appear in mutant adult organisms with defects in *Dmp52* were suppressed. Some of these *Dmp52* mutant phenotypes, such as cuticle deformations and brittle bristles, are caused by transcriptional defects during fly development and are, in many ways, quite homologous to some TTD manifestations [15],[16].

An enabling observation and important motivation for this work is evidence that overexpression of the human p8/TTDA gene in human fibroblasts derived from patients with TTD caused by a mutation in the XPD gene (mutant: XPD^R112H/R112H^) can suppress some of the phenotypes observed in this cell line [8]. However, because of the limitations of *in vitro*-cultured cell systems, defects that can be generated during development were not studied; the only phenotypes that could be analyzed were TFIIH levels and UV-irradiation sensitivity. Results obtained in cultured cells cannot always be extrapolated to a complete animal. TFIIH participates in three important and highly regulated functions during animal development that must be coordinated with differentiation programs at different developmental times. Mutations in TFIIH that reduce TFIIH functions do not necessarily have the same effects in different cell types. This is observed in humans, where some tissues or developmental processes (e.g., neurological defects) are preferentially affected depending on which subunit is mutated and where in the protein the amino acid change occurs [5],[6],[15],[16]. In this context, a recent work reported minimal differences in gene expression in proliferating fibroblasts from TTD, XPD, and normal donors, indicating that cultured cells do not recapitulate all the differences found in patients afflicted with different TFIIH-related syndromes [33]. Many of the phenotypes observed in flies with different *Dmp52* and *hay* alleles arise because of defects that accumulate during development. These defects can be partially corrected by the overexpression of Dmp8/TTDA; in other words, the suppression of *Dmp52* and *hay* mutations by Dmp8/TTDA is sufficient to allow the developmental program in a complex animal to run to completion.

The results presented here open the possibility that new treatments geared toward enhancing p8/TTDA function might stabilize TFIIH in patients with deficiencies of this DNA repair/transcription complex. This might be accomplished through the design of new drugs that enhance p8/TTDA function or by gene therapy strategies based on p8/TTDA overexpression. The effectiveness of either strategy may ultimately depend on resolving the three-dimensional structure of the interacting surfaces of p8/TTDA and other TFIIH subunits [34]. Mouse models, such as transgenic mice that carry XPD alleles known to cause TTD or XP/CS in humans, and which manifest some of the typical TTD or XP/CS phenotypes [35],[36], provide additional tools, making it possible to determine if the overexpression of p8/TTDA is able to rescue specific TFIIH-defective phenotypes.

Many cellular functions, including transcriptional activation, chromatin remodeling, and histone modification are mediated by multi-subunit protein complexes. Some of the subunits in these complexes are relatively small and have no known function, although mutations in some of these complex components have been linked to human diseases. It has been suggested that p8/TTDA may act as a kind of small chaperone protein to stabilize TFIIH [11], raising the possibility that, like p8/TTDA, some of these uncharacterized complex components may serve to maintain the stability and the steady state levels of the corresponding complexes. Thus, it will be important to determine whether proteins in other multi-subunit complexes possess p8/TTDA-like functions.

## Supporting Information

Figure S1 RT-PCR detection of *Dmp8/TTDA* transgene expression, Western analysis of Dmp8/TTDA-His protein expression in whole-fly extracts and immunostaining of salivary glands. (A) RT-PCR of transgenic flies overexpressing Dmp8/TTDA. Specific oligonucleotides designed against transcribed regions of the transgene not present in the endogenous *Dmp8/TTDA* mRNA were used to detect transgene expression in the TTDA1 and TTDA5 transgenic lines. Note that there no amplification product was obtained in the wild-type strain. (B) Semi-quantitative RT-PCR of *Dmp8/TTDA* mRNA from the wild-type line and a transgenic line (TTDA9) that overexpresses *Dmp8/TTDA* under the control of the HSP83 promoter. In this case, specific oligonucleotides that amplified both the endogenous and the transgenic *Dmp8/TTDA* mRNA were used. Amplification of *Rp49* mRNA from the same RT-PCR reactions was used as a control. The number of RT-PCR cycles is indicated in the figure. Note that at 30 cycles the endogenous *Dmp8/TTDA* mRNA is still difficult to detect. (C) Western blot of total protein extracts from adult transgenic flies expressing Dmp8/TTDA-H6 protein detected with an anti poly-histidine antibody. Molecular weight markers are indicated as M.M; wt indicates total proteins from a wild-type strain; TTDA9 indicates soluble (s) and precipitated (p) material from the transgenic line. (D) Immunostaining of salivary glands from a transgenic fly overexpressing recombinant Dmp8/TTDA-H6 protein using an anti-poly-histidine antibody. The staining was performed against salivary glands from non-irradiated larvae and larvae irradiated at 150 J/m^2^. Note that in the non-irradiated cells, a high proportion of Dmp8/TTDA is detected in the cytoplasm. In contrast, most of the Dmp8/TTDA signal in the irradiated cells is in the nuclei. This is in agreement with observations of the dynamics of p8/TTDA in human cultured cells after UV irradiation [11].
(4.52 MB TIF)

Figure S2 *mrn* ^3^ */mrn* ^3^ homozygous flies rescued by overexpression of Dmp8/TTDA exhibit a response to UV irradiation that is similar to that of the heterozygous *mrn* ^3^/+ line. Third instar larvae were irradiated and then allowed to develop to adults. Survival rate is indicated for each strain. The graph represents the results of at least three independent dose-response experiments for each genotype. The statistical analysis by ANOVA indicates a P value<0.001 for the parental strain (*red*, *e/red*, *e*) compared with the rescued homozygous (*mrn* ^3^ */mrn* ^3^) and the heterozygous (*mrn* ^3^/+) strains at 150 J/m^2^. The different genotypes are indicated in the figure.
(2.31 MB TIF)
