# Supplementary figures and images for: p8/TTDA Overexpression Enhances UV-Irradiation Resistance and Suppresses TFIIH Mutations in a Drosophila Trichothiodystrophy Model
Source: PLoS Genet. 2008 Nov 14;4(11):e1000253. doi: 10.1371/journal.pgen.1000253 (PMC2576456; doi:10.1371/journal.pgen.1000253)

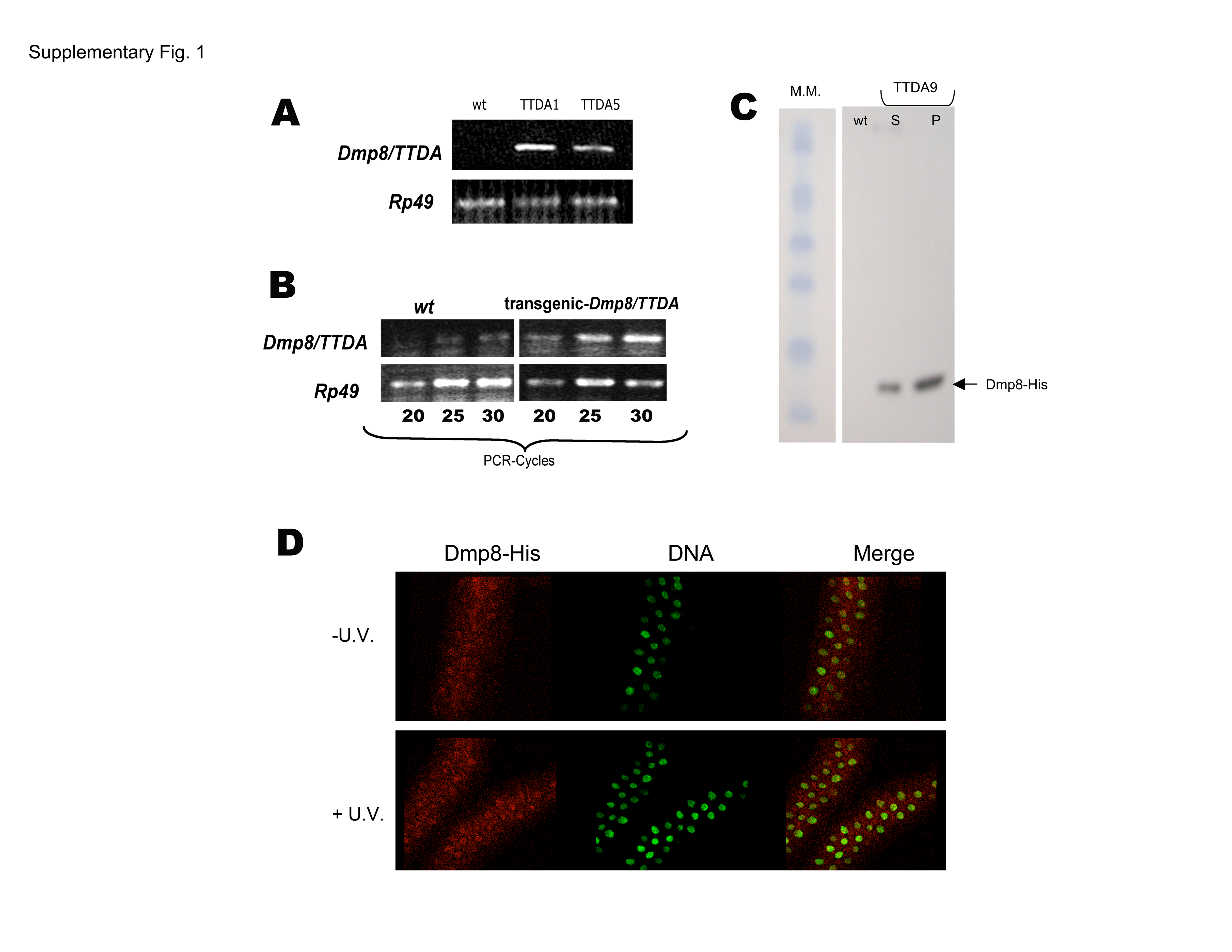

Supplement: Figure S1 — RT-PCR detection of Dmp8/TTDA transgene expression, Western analysis of Dmp8/TTDA-His protein expression in whole-fly extracts and immunostaining of salivary glands. (A) RT-PCR of transgenic flies overexpressing Dmp8/TTDA. Specific oligonucleotides designed against transcribed regions of the transgene not present in the endogenous Dmp8/TTDA mRNA were used to detect transgene expression in the TTDA1 and TTDA5 transgenic lines. Note that there no amplification product was obtained in the wild-type strain. (B) Semi-quantitative RT-PCR of Dmp8/TTDA mRNA from the wild-type line and a transgenic line (TTDA9) that overexpresses Dmp8/TTDA under the control of the HSP83 promoter. In this case, specific oligonucleotides that amplified both the endogenous and the transgenic Dmp8/TTDA mRNA were used. Amplification of Rp49 mRNA from the same RT-PCR reactions was used as a control. The number of RT-PCR cycles is indicated in the figure. Note that at 30 cycles the endogenous Dmp8/TTDA mRNA is still difficult to detect. (C) Western blot of total protein extracts from adult transgenic flies expressing Dmp8/TTDA-H6 protein detected with an anti poly-histidine antibody. Molecular weight markers are indicated as M.M; wt indicates total proteins from a wild-type strain; TTDA9 indicates soluble (s) and precipitated (p) material from the transgenic line. (D) Immunostaining of salivary glands from a transgenic fly overexpressing recombinant Dmp8/TTDA-H6 protein using an anti-poly-histidine antibody. The staining was performed against salivary glands from non-irradiated larvae and larvae irradiated at 150 J/m2. Note that in the non-irradiated cells, a high proportion of Dmp8/TTDA is detected in the cytoplasm. In contrast, most of the Dmp8/TTDA signal in the irradiated cells is in the nuclei. This is in agreement with observations of the dynamics of p8/TTDA in human cultured cells after UV irradiation [11]. (4.52 MB TIF) [file pgen.1000253.s001.tif]

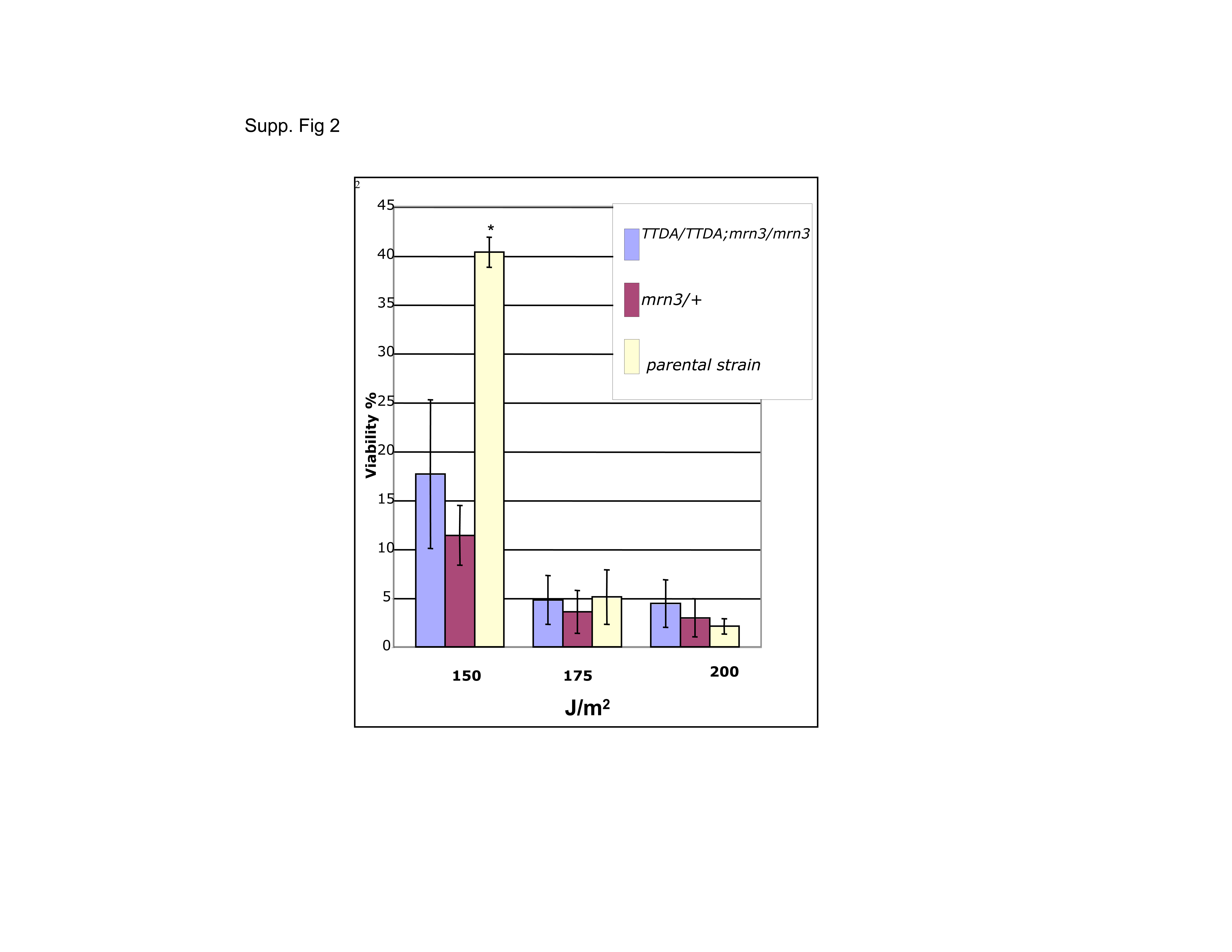

Supplement: Figure S2 — mrn 3 /mrn 3 homozygous flies rescued by overexpression of Dmp8/TTDA exhibit a response to UV irradiation that is similar to that of the heterozygous mrn 3/+ line. Third instar larvae were irradiated and then allowed to develop to adults. Survival rate is indicated for each strain. The graph represents the results of at least three independent dose-response experiments for each genotype. The statistical analysis by ANOVA indicates a P value<0.001 for the parental strain (red, e/red, e) compared with the rescued homozygous (mrn 3 /mrn 3) and the heterozygous (mrn 3/+) strains at 150 J/m2. The different genotypes are indicated in the figure. (2.31 MB TIF) [file pgen.1000253.s002.tif]
